# Trends in Depression Among Hospitalized Patients with Type 2 Diabetes in Spain (2017–2023): A Population-Based Analysis with a Focus on Sex Differences and In-Hospital Outcomes

**DOI:** 10.3390/jcm14113895

**Published:** 2025-06-01

**Authors:** Lucia Jiménez-Sierra, Natividad Cuadrado-Corrales, Valentín Hernández-Barrera, Rodrigo Jiménez-Garcia, Ana López-de-Andres, Javier de Miguel-Diez, Andrés Bodas-Pinedo, José J. Zamorano-León

**Affiliations:** 1 Department of Public Health and Maternal & Child Health, Faculty of Medicine, Universidad Complutense de Madrid, IdISSC, 28040 Madrid, Spain; lujime05@ucm.es (L.J.-S.); mariancu@ucm.es (N.C.-C.); anailo04@ucm.es (A.L.-d.-A.); abodas@ucm.es (A.B.-P.); josejzam@ucm.es (J.J.Z.-L.); 2 Preventive Medicine and Public Health Teaching and Research Unit, Health Sciences Faculty, Universidad Rey Juan Carlos, 28922 Madrid, Spain; valentin.hernandez@urjc.es; 3 Respiratory Care Department, Hospital General Universitario Gregorio Marañón, IiSGM, Universidad Complutense de Madrid, 28007 Madrid, Spain; javier.miguel@salud.madrid.org

**Keywords:** type 2 diabetes mellitus, depression, hospitalization, in-hospital mortality, sex differences, temporal trends

## Abstract

**Background/Objectives:** There is a growing body of evidence supporting a bidirectional relationship between depression and type 2 diabetes mellitus (T2DM). The coexistence of depression and T2DM has substantial clinical implications. However, there is little research looking at how these two conditions cluster together in people hospitalized with T2DM, the associated factors, and their effect on hospital outcomes. In this study, we aimed to assess temporal trends in depression prevalence among hospitalized patients with T2DM in Spain from 2017 to 2023. Additionally, we analyzed the association of variables such as gender, age, anxiety, obesity, alcohol and tobacco use, dementia, COVID-19 infection, and personality disorders in the presence of depression among hospitalized T2DM patients and the impact of these variables on in-hospital mortality (IHM). **Methods:** We conducted a population-based cohort study using the Spanish Hospital Discharge Registry (RAE-CMBD). Adults aged ≥ 40 years with a T2DM diagnosis were included. Depression was identified by using ICD-10 codes. Time trends were analyzed by using joinpoint regression. Multivariable logistic regression models were employed to identify factors associated with depression and in-hospital mortality (IHM), stratified by sex. **Results:** Among 4,597,668 hospitalizations with T2DM, 202,094 (4.39%) included a depression diagnosis. Depression prevalence increased slightly over time (APC: 1.09% in women and 0.98% in men). Women consistently showed higher prevalence (OR 3.21; 95% CI: 3.18–3.24). Age, anxiety, obesity, alcohol and tobacco use, and personality disorders were significantly associated with the presence of a code for depression, with notable sex differences. Among patients with T2DM and depression, IHM was significantly associated with older age, more comorbidities, COVID-19 infection, hypoglycemia, dementia, and female gender, whereas obesity and anxiety had a protective effect. **Conclusions:** From 2017 to 2023, the prevalence of depression in hospitalized patients with T2DM in Spain increased slightly, particularly among older women, highlighting the need for integrated mental health screening and management during hospitalization.

## 1. Introduction

According to the World Health Organization, depression is a common mental disorder affecting approximately 280 million people worldwide, which corresponds with an estimated prevalence of 5% of all adults [1]. However, the lifetime risk of depression is three-fold higher (15–18%), and its burden is projected to increase in the coming decades [2]. As with other chronic diseases, type 2 diabetes mellitus (T2DM) is associated with an increased risk of developing depression [3]. A recent meta-analysis indicates that individuals with T2DM are significantly more likely (1.5–1.7-fold) to experience major depressive disorder than those without diabetes [3]. This comorbidity has relevant clinical implications, as the presence of depression in patients with T2DM has been linked with higher hospitalization rates and increased healthcare costs [4,5]. In 2019, a meta-analysis showed that patients with T2DM and target organ complications had a higher likelihood of developing depression than those without complications (risk ratio of ~1.14; 95% CI: 1.07–1.21) [6]. However, some researchers have not identified a significant association between T2DM and depression after adjusting for comorbidities such as ischemic heart disease [7,8], suggesting the existence of shared risk factors that may partially explain this relationship.

In this context, the growing body of evidence supporting a bidirectional relationship between depression and T2DM becomes particularly relevant [9,10]. Early-onset T2DM slightly increases the risk of developing depression; conversely, individuals with depression are at greater risk of developing T2DM over time [10]. This complex interaction appears to be modulated by factors such as age, genetic predisposition, and the coexistence of other chronic conditions [11]. Various mechanisms have been proposed to explain this interrelation at the pathophysiological level. Depression is associated with both unhealthy lifestyle behaviors and persistent neuroendocrine alterations, including hyperactivity of the hypothalamic–pituitary–adrenal axis, elevated levels of cortisol and catecholamines, and increased proinflammatory cytokines [12]. These changes may contribute to insulin resistance and impaired glucose metabolism, favoring the onset or worsening of T2DM [12].

Several studies have shown that the bidirectional relationship between T2DM and depression is influenced by several comorbid factors, including anxiety, obesity, lifestyle habits (alcohol and tobacco use), dementia, and personality disorders. Anxiety is often comorbid with other psychiatric disorders shown to be associated with diabetes risk, such as depression [13]. Obesity is another relevant factor that may partly mediate the association of T2DM with depression [14,15,16,17,18]. Unhealthy behaviors such as alcohol consumption and smoking can also increase the risk of depression among patients with T2DM [19,20]. In the United Kingdom, between 2004 and 2019, according to data from the Clinical Practice Research Datalink, people with alcohol use disorders were approximately 50% more likely to be diagnosed with depression or anxiety after a diagnosis of T2DM [19]. Smoking has been strongly associated with greater depression prevalence among people with T2DM in epidemiological investigations conducted in several countries [20,21,22,23,24,25]. Co-occurring personality disorders and dementia have been linked with higher risks of both metabolic disease severity and depression, potentially compounding the diabetes–depression interplay [22,26,27,28].

The COVID-19 pandemic has further highlighted this complex interaction. Recent studies indicate that individuals with T2DM were at increased risk of anxiety and depression during this period [29,30]. 

The coexistence of depression and T2DM has substantial clinical implications. In patients with diabetes, depression has been associated with poorer glycemic control, lower treatment adherence, and an increased risk of cardiovascular complications and mortality [31,32]. Furthermore, sex differences play a key role in the prevalence and outcomes of both conditions. The scientific literature has consistently documented a higher frequency of depression in women with T2DM compared with men [33,34]. Khaledi et al. reported a depression prevalence of 34% in women versus 23% in men with T2DM [34]. Similarly, a national study conducted in Spain between 2001 and 2020 showed that the prevalence of diagnosed depression was approximately 3.32-fold higher in women with T2DM than in men [35]. The association between T2DM and depression has also been found to vary by sex, suggesting a potentially greater relative impact of diabetes on depression risk among women [14,36]. The proposed explanations include hormonal factors, sex-specific psychosocial stressors, and differences in help-seeking behaviors or clinical diagnosis patterns [14].

In this context, the aim of our study, based on nationwide administrative data, was to describe recent trends (2017–2023) in the prevalence of depression among hospitalized patients with T2DM in Spain. We also analyzed sex differences in depression prevalence according to hospital admission diagnoses, as well as the effect of coexisting depression on hospital outcomes in this population.

## 2. Materials and Methods

### 2.1. Study Design and Data Source

This population-based cohort study was conducted by using data from the Spanish Hospital Discharge Records, known as the Registry of Specialized Care Activity–Minimum Basic Data Set (RAE-CMBD). The data set comprises discharge records from all public health system hospitals spanning from 1 January 2017 to 31 December 2023.

The RAE-CMBD is a national administrative registry that systematically collects data on hospital admissions across Spain. All hospitals, both public and private, are required by law to send the data of patients who are discharged to the Spanish Ministry of Health. It is estimated that more than 95% of hospital discharges are registered by this system. In Spain, there is currently no other database that allows for the analysis of hospitalizations based on representative data from the entire country. The methodological framework has been previously described [37]. Diagnoses were coded according to the International Classification of Diseases, Tenth Revision (ICD-10).

### 2.2. Study Population 

This study included patients aged 40 years or older with a diagnosis of T2DM regardless of the diagnostic position in diagnosis fields. Patients with a diagnosis of type 1 diabetes mellitus were excluded.

ICD codes corresponding to depression were identified regardless of the diagnostic criteria to assess the association between T2DM and depression. The study population was subsequently stratified by the presence or absence of depression, and all analyses were further stratified by sex.

### 2.3. Study Variables

Comorbidities included in the Charlson Comorbidity Index (CCI) were assessed regardless of the diagnostic criteria, excluding diabetes and dementia, to evaluate the prevalence of depression in relation to common hospital admission diagnoses [38]. We additionally analyzed the presence of hypoglycemia, obesity, anxiety, specific personality disorders, intentional self-harm, suicide attempts, alcohol and tobacco use, all-cause dementia, Alzheimer’s dementia, vascular dementia, and COVID-19 infection. The complete list of the corresponding ICD-10 codes is provided in Table S1.

The hospital outcomes assessed included the length of hospital stay (LOHS), admission to the intensive care unit (ICU), and in-hospital mortality (IHM).

### 2.4. Ethical Considerations

Since the RAE-CMBD contains anonymized data and is publicly available upon request from the Spanish Ministry of Health, informed patient consent and ethics committee approval were not required according to the Spanish law [39,40].

### 2.5. Statistical Analysis

Descriptive statistics were used to summarize the data. Categorical variables are expressed as absolute frequencies and percentages, while continuous variables are presented as means with standard deviations for normally distributed data or medians with interquartile ranges for non-normally distributed data.

The time trends were analyzed by using appropriate statistical tests. Cochran–Mantel–Haenszel or Cochran–Armitage tests were applied to categorical variables, and linear regression *t*-tests or the Jonckheere–Terpstra test were used for continuous variables. Group comparisons were conducted by using Fisher’s exact test for categorical variables and Student’s *t*-test or the Wilcoxon rank-sum test for continuous variables, depending on the normal distribution of the data.

Log-linear joinpoint regression was used to identify periods with significant trend changes in depression rates among T2DM patients each year and estimate the annual percent change (APC) for each segment defined by the joinpoints [41]. The analyses were conducted by using Joinpoint Regression Program, version 4.0.4, and stratified by sex and age group.

Multivariable logistic regression models were employed to identify variables associated with the presence of a code for depression among patients with T2DM, accounting for potential sex differences. We used additional models to assess factors linked with IHM among men and women diagnosed with both T2DM and depression. The variables included in these models were those significant in any of the univariate analyses, namely, sex, age group, CCI, hypoglycemia, obesity, anxiety, personality disorders, intentionally self-inflicted injuries, suicide attempts, alcohol and tobacco use, Alzheimer’s dementia, vascular dementia, and COVID-19 infection. The results are presented as odds ratios (ORs) with 95% confidence intervals (CIs).

All statistical analyses were performed by using Stata, version 14 (StataCorp, College Station, TX, USA). Statistical significance was defined as a two-tailed *p*-value < 0.05.

## 3. Results

During the period 2017–2023, in Spain, a total of 4,597,668 discharges from public health system hospitals were recorded among patients aged ≥ 40 years with a diagnostic ICD-10 code corresponding to T2DM. Of these, 202,094 hospitalizations (4.39%) included an ICD-10 code for depression.

**Figure 1:**
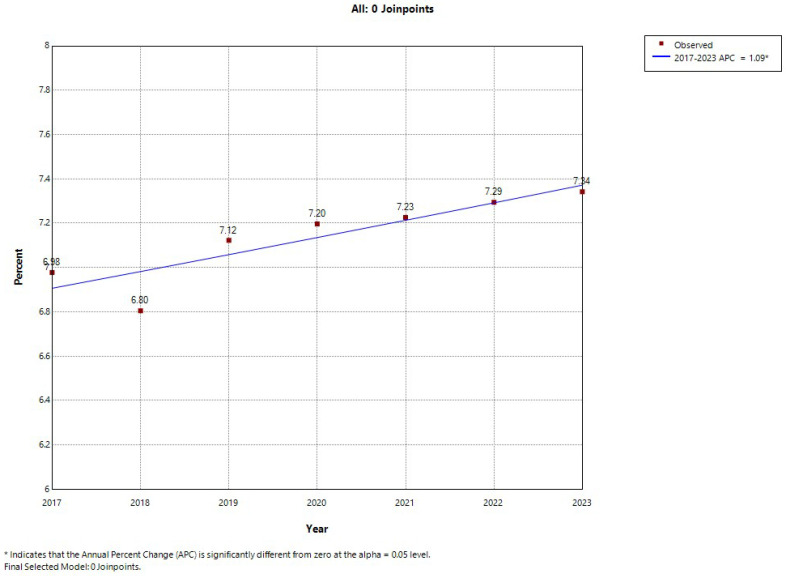
Joinpoint analysis of annual depression prevalence in women hospitalized with type 2 diabetes in Spain (2017–2023).

**Figure 2:**
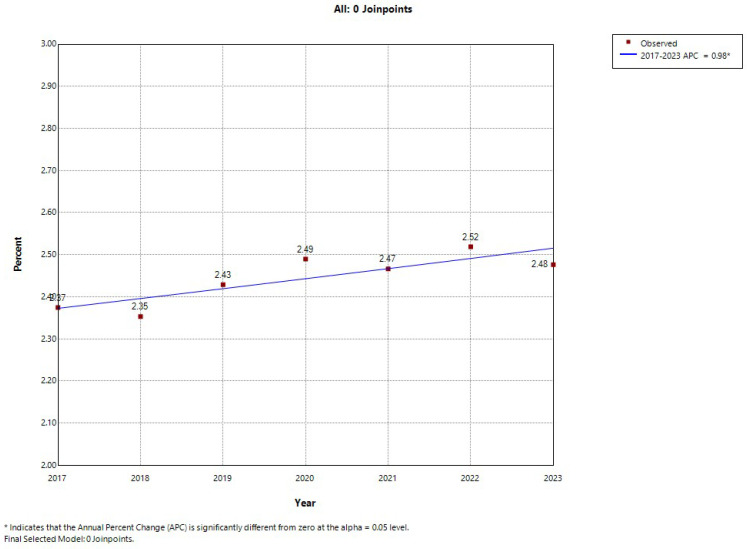
Joinpoint analysis of annual depression prevalence in men hospitalized with type 2 diabetes in Spain (2017–2023).

As observed in Figure 1 and Figure 2, the joinpoint regression analysis revealed sex trends in the annual prevalence of depression among patients with T2DM in Spain. In women, the overall prevalence increased significantly, with an APC of 1.09%. No significant point of inflection was found, showing a constant linear trend over time. When stratified by age group (Figure S1), women aged 40–64 years experienced a decrease in depression prevalence (APC: −1.16%; *p* < 0.05), while those aged 65–74 years showed a stable trend. No point of inflection was identified in either subpopulation. Among women aged 75–84 years, the prevalence rose between 2017 and 2020 (APC: 2.86%; *p* < 0.05). In the year 2020, a significant point of inflection was found, and from that year to 2023, the prevalence of depression stopped increasing and remained stable. The steepest increase, without any inflection point, was observed in women aged ≥ 85 years, with an APC of 3.24% (*p* < 0.05) from 2017 to 2023 (Figure S1).

In men, the overall trend also indicated a stable linear increase without any significant inflection point (APC: 0.98%; *p* < 0.05). In the analysis stratified by age group, as can be seen in Figure S2, in the 40–64-year group, depression prevalence decreased but not significantly from 2017 to 2019. An inflection point was detected that year, followed by a marked rise in the prevalence of depression from 2019 to 2023 (APC: 2.31%; *p* < 0.05). Men aged 65–74 and ≥85 years showed modest increases that were not significant, while a more pronounced increase was found in those aged 75–84 years (APC: 0.89%; *p* < 0.05) (Figure S2). Inflection points in the time prevalence of depression were found in none of these age groups.

As shown in Table 1, between 2017 and 2023, a significant increase was observed in the crude prevalence of depression among hospitalized patients with T2DM, rising from 4.33% in 2017 to 4.47% in 2023 (*p* < 0.001). Women consistently accounted for approximately two-thirds of the cases each year, with significantly higher prevalence than men (*p* < 0.001). The mean age of patients with T2DM and comorbid depression increased, from 74.77 years in 2017 to 76.02 years in 2023, as did the CCI, which rose from 0.95 to 1.00, respectively (all *p* < 0.001). A progressive increase in admissions to the ICU was also noted, from 4.79% to 5.6% (*p* < 0.001). The median LOHS remained stable at 6 days. IHM showed an upward trend from 2017 to 2020, peaking at 8.82% in 2020, followed by a progressive decline to 6.86% in 2023, although it did not return to pre-pandemic levels (*p* < 0.001).

As shown in Table 2, among the women hospitalized with T2DM, the mean age increased from 76.04 to 77.43 years, accompanied by a progressive rise in the CCI from 0.89 to 0.96 (both *p* < 0.001). Significant upward trends were observed in hypoglycemia (from 0.93% to 1.28%; *p* < 0.001) and anxiety (from 1.30% to 2.13%; *p* < 0.001), as well as specific personality disorders (from 1.01% to 1.40%; *p* = 0.002). The prevalence of all-cause dementia increased from 11.35% to 12.50% (*p* < 0.001). The prevalence of Alzheimer’s disease increased from 4.18% to 4.79%, (*p* = 0.012), while that of vascular dementia remained stable. The rate of intentional self-inflicted injuries fluctuated below 1%, and no significant trend was observed for suicide attempts. Alcohol consumption and tobacco use increased, with tobacco use rising from 9.62% to 13.97%. COVID-19 diagnoses appeared in 2020 (5.12%), peaked in 2022 (9.78%), and declined in 2023 (4.14%). ICU admissions rose modestly (from 4.25% to 4.91%; *p* = 0.035), while the median LOHS remained stable. IHM peaked in 2020 (8.74%) and decreased to 7.12% in 2023.

Among men hospitalized with T2DM (Table 3), the mean age rose from 71.99 to 73.13 years (*p* < 0.001), while the CCI remained stable. Anxiety doubled (from 0.96% to 2.08%; *p* < 0.001), intentional self-inflicted injuries rose modestly, and suicide attempts showed no significant trend. Alcohol use increased (13.52% to 15.72%; *p* < 0.001), as did tobacco use (39.23% to 40.74%; *p* = 0.004). Obesity and all-cause dementia showed significant upward trends, while the incidence of Alzheimer’s disease rose modestly, and that of vascular dementia remained stable. COVID-19 cases followed a similar pattern to those in women. ICU admissions rose (5.97% to 7%; *p* = 0.041), and IHM was the highest in 2020 (8.98%) and declined to 6.33% in 2023.

As indicated in Table S2, depression prevalence was consistently higher among women than men in all years of the study (all *p* < 0.001). Women were older (mean age: 76.78 vs. 72.69 years; *p* < 0.001) and had lower CCI scores (0.92 vs. 1.09; *p* < 0.001). Obesity and anxiety were more prevalent in women, while men showed higher rates of personality disorders, alcohol use (14.34% vs. 1.83%), and tobacco use (39.76% vs. 11.58%) (all *p* < 0.001). Suicide attempts were more common in men (0.18% vs. 0.06%; *p* = 0.026). Women had higher rates of all-cause and Alzheimer’s dementia (both *p* < 0.001). ICU admissions were more frequent in men (6.48% vs. 4.49%; <0.001), but IHM was similar between sexes (7.21% vs. 7.20%).

Shown in Table S3 are the IHM rates among men and women hospitalized with T2DM according to selected concomitant conditions and the presence of depression in Spain for the period 2017–2023. Women and men without depression had significantly higher IHM rates than those with this condition (women: 8.63% vs. 7.21% (*p* < 0.001); men: 7.51% vs. 7.2% (*p* = 0.002)). When the characteristics of those who died in hospital were compared, we found that men and women with depression had lower mean CCI scores than those without depression. Among women, IHM was lower across all age groups compared with those without depression, while in men, differences were significant only in the youngest and oldest groups. For both sexes, depression was associated with lower IHM in patients with hypoglycemia, obesity, anxiety, alcohol use, tobacco use, and any type of dementia. COVID-19 cases among depressed patients also showed lower IHM. 

The multivariable analyses (Table 4) showed that the probability of depression increased over time in both sexes, particularly in 2022 and 2023. Older age in women was associated with a higher depression risk, whereas the CCI was inversely associated in both sexes. Personality disorders, self-inflicted injuries, and suicide attempts were strongly associated with depression, especially in men. Anxiety showed a protective effect, mainly in women. Alcohol use increased the likelihood of depression in both sexes, while tobacco use had opposite effects (a protective effect in men and a higher risk in women). In the T2DM population, after adjusting for age and all the comorbid conditions, women were 3.21-fold more likely to have a code for depression in their discharge report than men (OR 3.21; 95% CI 3.18–3.24).

Regarding IHM, older age and a higher CCI were the strongest predictors. COVID-19 diagnosis was associated with a two-fold increase in IHM. Obesity had a protective effect, while hypoglycemia and dementia increased mortality. Anxiety was associated with lower IHM, particularly in men. Being a woman was significantly associated with IHM (OR 1.11; 95% CI 1.07–1.16).

## 4. Discussion

This nationwide analysis of hospital data in Spain provides a comprehensive overview of temporal trends and clinical characteristics associated with depression among patients hospitalized with T2DM between 2017 and 2023. Depression was coded in 4.39% of all hospitalizations for T2DM, and its overall prevalence showed a slight upward trend over time, with consistently higher rates being observed in women compared with men. IHM among individuals with T2DM and depression increased during the COVID-19 pandemic (2020−2021), and sex-related differences in IHM were observed in this population.

### 4.1. Time Trends in Prevalence of Depression Among Hospitalized Adults with Type 2 Diabetes Mellitus (T2DM) 

The prevalence of depression among patients with diabetes has increased over time in other countries, in line with our investigation [42]. In a previous study in Spain, an upward trend in the prevalence of depression among hospitalized patients with T2DM between 2011 and 2015 was reported [43]; however, more recent findings suggest a decline in prevalence since 2016 [35]. In our study, the age-stratified analysis revealed a decreasing trend in depression prevalence among middle-aged women (40–64 years), whereas older women, particularly those aged ≥ 85 years, experienced the most pronounced increase. Among men, the sharpest rise was observed in those aged 75–84 years, with a recent increase also detected in the 40–64 age group after 2019. The rise in depression among older adults, especially women ≥ 85 years and men aged 75–84, may be explained by factors such as higher multimorbidity, social isolation, loss of independence, and reduced cognitive reserve [44,45,46]. 

As far as we know, there are no international studies based on hospital data on the prevalence of depression in subjects with T2DM over time. However, the prevalence and incidence of depression among people suffering from T2DM have been reported in several studies based on registries and consecutive cross-sectional studies, with discordant results [34,42,47,48,49,50].

Dibeto et al., using electronic medical records from the UK and the USA and based on two cohorts of approximately 1.4 million adults with an incident of T2DM diagnosed between 2006 and 2017, reported that depression prevalence in the UK/USA rose from 29% in 2006 to 43% in 2017, with a similar increasing trend for incidence rates [42]. The authors of a meta-analysis of observational studies agree with this time trend, reporting that the prevalence of depression among people with T2DM has increased from 20% (results published in 2007) to 32%, based on studies published in 2018 [34]. In the UK, among 108,588 people with T2DM registered in Clinical Practice Research Datalink, a diagnostic code of depression in the previous 12 months or the presence of ≥3 prescriptions for antidepressants in the previous 12 months was found in 10.2% of individuals in the year 2004, which increased to 12.4% in 2014 [48].

However, a cohort study in the USA based on National Health and Nutrition Examination Survey (NHANES) 2005–2018 data showed that the prevalence of depression among individuals with T2DM remained stable between 2005 and 2018, both from an overall point of view and a gender–age subgroup perspective [47]. In Norway, a study on data from the population-based Trøndelag Health Study (HUNT) including three waves, i.e., HUNT2 (1995–1997), HUNT3 (2006–2008), and HUNT4 (2017–2019), showed that depressive symptom prevalence showed an overall decline in the first period yet increased slightly in women and remained largely unchanged in men throughout the study period [49]. Such trend resulted in an overall decrease in depressive symptoms in men with diabetes and relative symptom stability in women with this condition [49]. Similarly, a decline in depressive symptom rates was observed, from 2001 to 2015, in a population-based sample of Mexican adults aged ≥ 50 years with diabetes [50].

Possible variations in data sources, participant characteristics, sample sizes, study sites, time periods, and methods of assessing depression and T2DM make it difficult to compare the results of these studies.

### 4.2. Variables Associated with the Presence of Depression Among Hospitalized Adults with Type 2 Diabetes Mellitus (T2DM) 

Depression prevalence was 3.21-fold higher in women than in men with T2DM, consistently with all previous studies in which a greater burden of depressive disorders among women with T2DM has been constantly reported [14,18,33,34,35,36,42,43]. Several factors may contribute to these sex differences. Higher rates of depression in women have been attributed to a combination of biological, psychological, and social factors, including hormonal fluctuations, greater caregiving responsibilities, a higher propensity to internalize symptoms, and higher prevalence of obesity [14,18,33,34,35,36,42,43]. In line with these findings, our study showed that obesity was associated with higher odds of depression in both sexes.

The association between obesity and depression in patients with diabetes is well documented in the scientific literature [15,16,17,24,47]. A study on data from the 2005–2014 NHANES showed that obesity accounted for 28.6% of the risk of depression among individuals with diabetes, indicating a strong connection between the two conditions [16]. Moreover, a recent systematic review identified that major depression with atypical and somatic features is consistently associated with an increased risk of both obesity and diabetes, underscoring the tight interconnection among these disorders [17]. The association among T2DM, depression, and obesity is believed to be due to overlapping biological links, such as elevated cytokine levels, impaired neurotransmitter metabolism due to insulin deficiency, and hyperactivity of the hypothalamic–pituitary–adrenal (HPA) axis [51]. These findings highlight the importance of integrating mental health into the comprehensive care of patients with both obesity and diabetes.

Although the overall comorbidity burden (as measured according to the CCI) showed an inverse association with depression, specific conditions such as hypoglycemia, dementia, and certain mental health disorders (including personality disorders and suicide attempts) were significantly associated with depression in patients with T2DM. Hypoglycemia has been linked with increased emotional distress and depressive symptoms, potentially due to the fear of recurrent episodes and glucose fluctuations that affect brain function [52]. Similarly, both Alzheimer’s and vascular dementia have been independently associated with depressive disorders in older adults with diabetes [26]. 

Mental disorders, particularly personality disorders and suicidal behaviors, are known to be associated with depression in patients with chronic diseases, including diabetes [28,52,53].

The relationship between anxiety and depression in individuals with diabetes is complex and multifactorial. In our study, anxiety showed an inverse association with depression. It has been suggested that among those with diabetes, a certain level of anxiety may act as a monitoring mechanism, promoting treatment adherence and complication prevention, which in turn may reduce the likelihood of depression [13,54]. Naicker et al., in the Norwegian HUNT study, found that depressive symptoms were associated with poorer health behaviors (lower dietary adherence: OR = 0.20 [95% CI: 0.06–0.68]; physical inactivity: OR = 1.69 [95% CI: 1.37–2.72]), whereas anxiety was linked with healthier self-care behaviors, such as higher vegetable intake (OR = 1.66 [95% CI: 1.02–2.73]) [13].

Lifestyle factors such as alcohol and tobacco use were positively associated with depression in patients with T2DM. Cook et al., in a study conducted in England, reported that individuals with alcohol use disorders had more than double the rate of depression following a diagnosis of T2DM compared with those without alcohol use disorder [19]. 

Similarly, the researchers of a systematic review identified smoking as a risk factor for the development of T2DM, suggesting that tobacco use may impact both metabolic and mental health outcomes in this population [20]. Wu et al. analyzed the association of substance use (alcohol, tobacco, and drugs) and related mental disorders with T2DM adults by using electronic health records in the USA. People with prevalent T2DM and substance use disorders had 2.22-fold higher odds of a prevalent mood disorder (including depression), 1.87-fold higher odds of anxiety, and 3.65-fold higher odds of a personality disorder than people without substance use disorder [22]. One potential explanation is that alcohol and tobacco use may disrupt neurochemical and hormonal regulation, thereby contributing to depressive symptoms. Additionally, these behaviors may impair diabetes self-management and increase psychological stress, further predisposing individuals to depression [19,20].

In our study, no significant association was found between the COVID-19 pandemic and the presence of depression in patients with T2DM. 

Most international studies suggest that the COVID-19 pandemic has adversely affected mental health among patients with T2DM [29,30,55,56]. Chao et al., in a US cohort of older adults with T2DM, reported an increase in depression prevalence from 19.3% before the pandemic to 30.4% during the pandemic [30]. In a global survey of 1829 diabetes nurses from 27 countries, 48% of the respondents reported seeing some adverse effects of COVID-19 on the psychological and physical risks of people with diabetes. These included an increase in depression (893 [49%]), anxiety (1486 [82%]), and diabetes distress (1189 [65%]) [56]. 

Several studies have shown that prolonged exposure to stressors such as those experienced due to social isolation, healthcare avoidance due to concern of being infected or the will to help reduce strain on healthcare services already overburdened, and the loss of relatives or friends increase the risk of depression and anxiety and post-traumatic stress disorders in people with diabetes [55,56,57,58]. Recurrent lockdowns and public health measures throughout the pandemic have reduced access to diabetes care, as many patients delayed or canceled medical appointments or routine control visits and experienced interruptions in their diabetes management due to health services being highly limited due to the pandemic [57,58]. Furthermore, self-management, as well as lifestyle behaviors, was negatively affected by the pandemic, with some patients even having difficulties accessing diabetes medications. All these facts exacerbate psychological distress, alter emotional wellbeing, and increase mental disorders in individuals with T2DM [55,56,57,58,59].

These findings suggest that the pandemic may have worsened the mental health of the T2DM population in Spain despite no significant association being found in our study. In the future, researchers should delve into this issue, focusing on not only the hospital environment but also primary care.

### 4.3. Variables Associated with In-Hospital Mortality Among Adults with Type 2 Diabetes Mellitus (T2DM) Hospitalized with Concomitant Depression

In our study, women with T2DM and depression were at higher risk of IHM than men. This finding aligns with previous evidence showing sex-related differences in clinical outcomes among patients with diabetes and comorbid mental disorders. A recent population-based study on NHANES 2005–2018 data showed that depression significantly increased all-cause mortality in people with T2DM, with a more pronounced effect in women, particularly in older age groups [47]. Similarly, Wu et al. reported that women with diabetes were at higher risk of mortality and macrovascular complications associated with depression than men [60]. Although women with T2DM typically present with lower comorbidity indices and are less frequently admitted to ICUs, they tend to be older at the time of hospitalization, a factor that may contribute to the increased mortality risk observed in this group [61].

Among the main risk factors associated with IHM in patients with T2DM and depression in our study population were older age, comorbidity, hypoglycemia, dementia, and alcohol use, as previously reported in the literature [35,43,62]. In contrast, anxiety and obesity were associated with a protective effect. Huang et al., using Taiwan’s National Health Insurance Database, found that patients with diabetes and comorbid anxiety had a higher five-year survival rate than those without anxiety disorders, suggesting a protective effect of anxiety on mortality outcomes [63]. Moreover, the so-called “obesity paradox”, i.e., the existence of better clinical outcomes in some overweight patients, has been debated in various clinical settings. Possible explanations include greater energy and metabolic reserves, more intensive medical follow-up, and reverse causality, whereby weight loss may reflect underlying poor health rather than causing it [62,64,65].

Nevertheless, COVID-19 infection was associated with increased IHM in patients with both T2DM and depression [66,67]. A plausible explanation is that the combination of these comorbidities may exacerbate patient vulnerability, increasing the risk of adverse outcomes during hospitalization [68].

### 4.4. Strengths and Limitations

The main strength of our study is its population-based approach, which used a stable, exhaustive, valid, and reliable database over a period of seven years. Therefore, we identified enough cases to be able to stratify by both demographic and clinical variables and build robust and accurate multivariable models. It is noteworthy that the RAE-CMBD is periodically evaluated to ensure its quality and has been used in previous epidemiological studies [35,37,43].

The findings of this study should be interpreted considering several limitations inherent in the design and data source. First, depression was identified exclusively based on ICD-10 codes recorded in the RAE-CMBD, which may underestimate its true prevalence due to the missing, undiagnosed, uncoded cases or coding errors. It is possible that the physicians who fulfill the discharge procedure only record the most severe cases of depression, producing a selection bias; therefore, the detected prevalence would be lower than that in the out-of-hospital population. Studies conducted in other countries have shown that the under-coding of depression in administrative data is frequent, even though most coded cases are genuine, resulting in very high specificity (often ≥ 95%) and a positive predictive value but low sensitivity [69,70,71]. Second, data on the onset, severity, or treatment of depression were not available, preventing differentiation between mild and severe forms of depression or between preexisting and newly diagnosed depression conditions, due to limitations of the ICD-10 information recorded in our database. Third, the analysis was based on hospitalization episodes rather than unique patients, and the lack of longitudinal follow-up may introduce bias due to readmissions. Fourth, as for depression, key clinical variables for diabetes, such as glycemic control, duration, chronic complications, or treatments, were not available, limiting adjustment for confounding factors. Fifth, patients included in this analysis were restricted to those aged 40 years and older based on epidemiological considerations, given that the prevalence of T2DM below this age in Spain is very low [72]. Furthermore, younger populations are at higher risk of diagnostic misclassification with type 1 diabetes, latent autoimmune diabetes in adults (LADA), or other types of diabetes [73,74]. Therefore, our findings are not generalizable to younger patients or those managed in outpatient settings. 

### 4.5. Recommendations

There is a growing consensus that all patients hospitalized with T2DM should be routinely screened for depression and anxiety [42,75,76,77]. In this way, the early identification of people at risk would be achieved, which would in turn allow for the rapid initiation of interventions such as psychosocial support or psychiatric treatment depending on the characteristics of the disorder and the patient [75]. Previous studies have indicated that the implementation of screening for depression at the time of hospital admission for patients with T2DM is feasible and acceptable for clinicians and patients and helps to ensure that the psychological needs of patients with T2D are addressed during hospitalization, thus facilitating diabetes management and recovery [76]. The results of our investigation can help to identify those with T2DM that are more likely to benefit from screening for mental conditions.

Future research should be aimed at providing an understanding of how depression interacts with T2DM and other comorbidities. In addition, given the complexity of the roles of comorbidities in the interplay of diabetes and depression, detailed evaluations of the bidirectional association of these conditions by ethnicity, age group, and sex are crucial [78].

## 5. Conclusions

In conclusion, this nationwide study conducted between 2017 and 2023 revealed a progressive increase in coded depression among hospitalized patients with T2DM in Spain, with higher prevalence among women and older age groups. Significant sex-based clinical differences were identified. Depression was associated with lower IHM, independently of age, comorbidity, and preexisting clinical characteristics at admission. While no significant association was found between the COVID-19 pandemic and the trends in the presence of depression in patients with T2DM, COVID-19 infection was linked with increased IHM. 

## Figures and Tables

**Table 1:** Characteristics of patients hospitalized in Spain with type 2 diabetes suffering concomitant depression by year (2017–2023).

	2017	2018	2019	2020	2021	2022	2023	Time Trend Analysis *p*
T2DM, n	620,395	650,396	663,794	611,899	653,517	688,486	709,181	
Prevalence of depression, n (%)	26,889 (4.33)	27,446 (4.22)	29,136 (4.39)	27,091 (4.43)	28,928 (4.43)	30,932 (4.49)	31,672 (4.47)	<0.001
Men, n (%)	8460 (31.46)	8888 (32.38)	9388 (32.22)	8963 (33.08)	9481 (32.77)	10,175 (32.89)	10,382 (32.78)	<0.001
Women, n (%)	18,429 (68.54)	18,558 (67.62)	19,748 (67.78)	18,128 (66.92)	19,447 (67.23)	20,757 (67.11)	21,290 (67.22)	
Age, mean (SD)	74.77 (11.07)	74.99 (11.11)	75.19 (11.1)	75.48 (11.14)	75.48 (11.17)	76.05 (11.14)	76.02 (11.26)	<0.001
CCI, mean (SD)	0.95 (0.91)	0.95 (0.91)	0.97 (0.92)	0.97 (0.92)	0.98 (0.93)	0.99 (0.93)	1 (0.92)	<0.001
Admission to ICU, n (%)	1289 (4.79)	1368 (4.98)	1481 (5.08)	1405 (5.19)	1449 (5.01)	1621 (5.24)	1773 (5.6)	<0.001
LOHS, median (IQR)	6 (8)	6 (7)	6 (7)	6 (8)	6 (8)	6 (7)	6 (7)	0.089
IHM, n (%)	1717 (6.39)	1776 (6.47)	1933 (6.63)	2389 (8.82)	2251 (7.78)	2328 (7.53)	2173 (6.86)	<0.001

T2DM: type 2 diabetes mellitus; CCI: Charlson Comorbidity Index; ICU: intensive care unit; IHM: in-hospital mortality; LOHS: length of hospital stay; IQR: interquartile range.

**Table 2:** Prevalence of depression, distributed by age, clinical characteristics, and in-hospital outcomes among women hospitalized with type 2 diabetes in Spain in 2017–2023.

	2017	2018	2019	2020	2021	2022	2023	Time Trend Analysis *p*
Age, mean (SD)	76.04 (10.75)	76.25 (10.75)	76.38 (10.84)	76.81 (10.8)	76.9 (10.87)	77.47 (10.82)	77.43 (10.98)	<0.001
CCI, mean (SD)	0.89 (0.88)	0.88 (0.88)	0.91 (0.89)	0.91 (0.89)	0.93 (0.89)	0.94 (0.9)	0.96 (0.9)	<0.001
Hypoglycemia, n (%)	171 (0.93)	253 (1.36)	249 (1.26)	263 (1.45)	267 (1.37)	289 (1.39)	273 (1.28)	<0.001
Obesity, n (%)	4176 (22.66)	4035 (21.74)	4617 (23.38)	4236 (23.37)	4771 (24.53)	4829 (23.26)	4787 (22.48)	<0.001
Anxiety, n (%)	240 (1.3)	255 (1.37)	319 (1.62)	320 (1.77)	395 (2.03)	472 (2.27)	453 (2.13)	<0.001
Specific personality disorders, n (%)	187 (1.01)	185 (1)	225 (1.14)	190 (1.05)	231 (1.19)	249 (1.2)	297 (1.4)	0.002
Intentionally self-inflicted injuries, n (%)	84 (0.46)	167 (0.9)	163 (0.83)	149 (0.82)	140 (0.72)	131 (0.63)	159 (0.75)	<0.001
Suicide attempts, n (%)	15 (0.08)	11 (0.06)	9 (0.05)	9 (0.05)	7 (0.04)	14 (0.07)	17 (0.08)	0.439
Alcohol, n (%)	273 (1.48)	281 (1.51)	372 (1.88)	347 (1.91)	398 (2.05)	417 (2.01)	402 (1.89)	<0.001
Tobacco use, n (%)	1773 (9.62)	1884 (10.15)	2211 (11.2)	2003 (11.05)	2278 (11.71)	2662 (12.82)	2975 (13.97)	<0.001
All-cause dementia, n (%)	2091 (11.35)	2139 (11.53)	2346 (11.88)	2289 (12.63)	2394 (12.31)	2678 (12.9)	2661 (12.5)	<0.001
Alzheimer’s dementia, n (%)	771 (4.18)	826 (4.45)	914 (4.63)	886 (4.89)	871 (4.48)	1002 (4.83)	1019 (4.79)	0.012
Vascular dementia, n (%)	412 (2.24)	365 (1.97)	445 (2.25)	407 (2.25)	448 (2.3)	484 (2.33)	490 (2.3)	0.247
COVID-19 infection, n (%)	0 (0)	0 (0)	0 (0)	929 (5.12)	1525 (7.84)	2030 (9.78)	881 (4.14)	<0.001
Admission to ICU, n (%)	784 (4.25)	828 (4.46)	880 (4.46)	828 (4.57)	834 (4.29)	924 (4.45)	1046 (4.91)	0.035
LOHS, median (IQR)	6 (7)	6 (7)	6 (7)	6 (7)	6 (7)	6 (7)	6 (7)	0.102
IHM, n (%)	1152 (6.25)	1174 (6.33)	1280 (6.48)	1584 (8.74)	1527 (7.85)	1602 (7.72)	1516 (7.12)	<0.001

CCI: Charlson Comorbidity Index; ICU: intensive care unit; IHM: in-hospital mortality; LOHS: length of hospital stay; IQR: interquartile range.

**Table 3:** Prevalence of depression, distributed by age, clinical characteristics, and in-hospital outcomes among men hospitalized with type 2 diabetes in Spain in 2017–2023.

	2017	2018	2019	2020	2021	2022	2023	Time Trend Analysis *p*
Age, mean (SD)	71.99 (11.26)	72.36 (11.4)	72.7 (11.25)	72.79 (11.33)	72.55 (11.21)	73.15 (11.22)	73.13 (11.26)	<0.001
CCI, mean (SD)	1.07 (0.95)	1.09 (0.96)	1.1 (0.98)	1.09 (0.97)	1.08 (0.99)	1.09 (0.97)	1.08 (0.96)	0.494
Hypoglycemia, n (%)	96 (1.13)	108 (1.22)	112 (1.19)	118 (1.32)	112 (1.18)	157 (1.54)	115 (1.11)	0.097
Obesity, n (%)	1143 (13.51)	1249 (14.05)	1336 (14.23)	1330 (14.84)	1578 (16.64)	1664 (16.35)	1649 (15.88)	<0.001
Anxiety, n (%)	81 (0.96)	107 (1.2)	120 (1.28)	132 (1.47)	156 (1.65)	167 (1.64)	216 (2.08)	<0.001
Specific personality disorders, n (%)	137 (1.62)	125 (1.41)	149 (1.59)	171 (1.91)	178 (1.88)	180 (1.77)	192 (1.85)	0.086
Intentionally self-inflicted injuries, n (%)	33 (0.39)	84 (0.95)	57 (0.61)	57 (0.64)	76 (0.8)	66 (0.65)	71 (0.68)	<0.001
Suicide attempts, n (%)	13 (0.15)	20 (0.23)	25 (0.27)	14 (0.16)	14 (0.15)	16 (0.16)	18 (0.17)	0.395
Alcohol, n (%)	1144 (13.52)	1200 (13.5)	1264 (13.46)	1214 (13.54)	1427 (15.05)	1543 (15.16)	1632 (15.72)	<0.001
Tobacco use, n (%)	3319 (39.23)	3445 (38.76)	3864 (41.16)	3509 (39.15)	3717 (39.2)	4054 (39.84)	4230 (40.74)	0.004
All-cause dementia, n (%)	793 (9.37)	905 (10.18)	875 (9.32)	893 (9.96)	980 (10.34)	1092 (10.73)	1067 (10.28)	0.01
Alzheimer’s dementia, n (%)	229 (2.71)	262 (2.95)	251 (2.67)	191 (2.13)	263 (2.77)	279 (2.74)	294 (2.83)	0.028
Vascular dementia, n (%)	170 (2.01)	198 (2.23)	217 (2.31)	211 (2.35)	226 (2.38)	249 (2.45)	237 (2.28)	0.57
COVID-19 infection, n (%)	0 (0)	0 (0)	0 (0)	472 (5.27)	703 (7.41)	1054 (10.36)	448 (4.32)	<0.001
Admission to ICU, n (%)	505 (5.97)	540 (6.08)	601 (6.4)	577 (6.44)	615 (6.49)	697 (6.85)	727 (7)	0.041
LOHS, median (IQR)	6 (8)	6 (8)	6 (8)	6 (8)	6 (8)	6 (7)	6 (7)	0.091
IHM, n (%)	565 (6.68)	602 (6.77)	653 (6.96)	805 (8.98)	724 (7.64)	726 (7.14)	657 (6.33)	<0.001

CCI: Charlson Comorbidity Index; ICU: intensive care unit; IHM: in-hospital mortality; LOHS: length of hospital stay; IQR: interquartile range.

**Table 4:** The multivariable analysis of the factors associated with the presence of depression among men and women hospitalized with type 2 diabetes and the factors associated with in-hospital mortality among men and women with type 2 diabetes and concomitant depression in Spain in 2017–2023.

		Presence of Depression			IHM in Patients with T2DM and Depression		
		Men	Women	Both Sexes	Men	Women	Both Sexes
		OR (95%CI)	OR (95%CI)	OR (95%CI)	OR (95%CI)	OR (95%CI)	OR (95%CI)
Year of admission	2017	1	1	1	1	1	1
	2018	0.99 (0.96–1.02)	0.98 (0.96–1)	0.99 (0.97–1)	0.99 (0.88–1.12)	1 (0.92–1.09)	0.99 (0.93–1.07)
	2019	1.02 (0.99–1.05)	1.04 (1.01–1.06)	1.03 (1.01–1.05)	1.01 (0.9–1.14)	1 (0.92–1.09)	1 (0.94–1.07)
	2020	1.04 (1.01–1.07)	1.05 (1.03–1.07)	1.05 (1.03–1.07)	1.28 (1.14–1.44)	1.32 (1.22–1.43)	1.31 (1.22–1.4)
	2021	1.03 (1–1.06)	1.06 (1.04–1.08)	1.05 (1.03–1.07)	1.06 (0.94–1.19)	1.14 (1.05–1.24)	1.11 (1.04–1.19)
	2022	1.05 (1.02–1.08)	1.07 (1.05–1.1)	1.07 (1.05–1.09)	0.92 (0.82–1.04)	1.05 (0.97–1.14)	1.01 (0.94–1.08)
	2023	1.03 (1–1.06)	1.08 (1.06–1.1)	1.07 (1.05–1.08)	0.87 (0.77–0.98)	1 (0.92–1.08)	0.96 (0.9–1.02)
Age group	40–64 years	1	1	1	1	1	1
	65–74 years	0.91 (0.9–0.93)	1.16 (1.14–1.19)	1.04 (1.02–1.05)	1.47 (1.32–1.64)	1.53 (1.37–1.7)	1.49 (1.38–1.6)
	75–84 years	0.91 (0.89–0.93)	1.15 (1.13–1.17)	1.03 (1.02–1.05)	2.12 (1.91–2.35)	2.41 (2.18–2.67)	2.25 (2.09–2.42)
	≥85 years	0.99 (0.96–1.02)	0.98 (0.96–1)	0.93 (0.91–0.94)	3.3 (2.96–3.68)	4.11 (3.71–4.56)	3.75 (3.49–4.03)
CCI		0.97 (0.96–0.98)	0.94 (0.93–0.95)	0.95 (0.95–0.95)	1.41 (1.37–1.45)	1.46 (1.43–1.49)	1.44 (1.42–1.47)
Hypoglycemia		1.23 (1.14–1.32)	0.99 (0.95–1.04)	1.06 (1.02–1.11)	1.45 (1.16–1.81)	1.17 (0.99–1.38)	1.26 (1.1–1.44)
Obesity		1.13 (1.1–1.15)	1.25 (1.24–1.27)	1.22 (1.21–1.23)	0.67 (0.61–0.74)	0.84 (0.79–0.88)	0.79 (0.76–0.83)
Anxiety		0.79 (0.74–0.84)	0.29 (0.28–0.31)	0.36 (0.35–0.37)	0.6 (0.42–0.85)	0.82 (0.68–0.98)	0.76 (0.64–0.89)
Specific personality disorders		5.73 (5.37–6.11)	2.85 (2.7–3.02)	3.77 (3.61–3.94)	0.8 (0.59–1.09)	0.69 (0.51–0.92)	0.74 (0.59–0.91)
Intentionally self-inflicted injuries		1.41 (1.28–1.55)	1.04 (0.98–1.12)	1.14 (1.08–1.21)	1.8 (1.3–2.48)	0.62 (0.45–0.86)	0.94 (0.75–1.18)
Suicide attempts		8.52 (6.84–10.62)	4 (3.06–5.22)	6.47 (5.44–7.69)	0.62 (0.23–1.7)	1.11 (0.35–3.55)	0.82 (0.38–1.76)
Alcohol		1.29 (1.26–1.32)	1.24 (1.19–1.29)	1.26 (1.23–1.28)	1.13 (1.02–1.24)	1.11 (0.91–1.35)	1.14 (1.04–1.24)
Tobacco use		0.94 (0.93–0.96)	1.19 (1.17–1.21)	1.03 (1.02–1.04)	0.84 (0.79–0.9)	0.9 (0.82–0.98)	0.86 (0.82–0.91)
Alzheimer’s dementia		1.44 (1.37–1.51)	1.1 (1.07–1.13)	1.16 (1.13–1.18)	1.46 (1.26–1.69)	1.63 (1.51–1.76)	1.59 (1.48–1.7)
Vascular dementia		1.86 (1.77–1.96)	1.41 (1.36–1.47)	1.54 (1.5–1.59)	1.23 (1.05–1.45)	1.06 (0.94–1.19)	1.11 (1.01–1.22)
COVID-19 infection		1 (0.96–1.04)	0.97 (0.95–1)	0.98 (0.96–1.01)	2.11 (1.86–2.38)	1.97 (1.81–2.15)	2.01 (1.87–2.16)
Sex	Men	NA	NA	1	NA	NA	1
	Women	NA	NA	3.21 (3.18–3.24)	NA	NA	1.11 (1.07–1.16)

IHM: in-hospital mortality; T2DM: type 2 diabetes; CCI: Charlson Comorbidity Index; OR: odds ratio; CI: confidence interval; NA: not available.

## Data Availability

According to the contract signed with the Spanish Ministry of Health and Social Services, which provided access to the databases from the Spanish National Hospital Database, we cannot share the databases with any other investigator, and we have to destroy the databases once the investigation has concluded. Consequently, we cannot upload the databases to any public repository. However, any investigator can apply for access to the databases by filling out the questionnaire available at https://www.sanidad.gob.es/estadEstudios/estadisticas/estadisticas/estMinisterio/SolicitudCMBD.htm (accessed on 16 December 2024). All other relevant data are included in the paper.

## Supplementary Materials

The following supporting information can be downloaded at: https://www.mdpi.com/article/10.3390/jcm14113895/s1. Table S1: Diagnosis analyzed with their corresponding ICD-10 codes; Table S2: Prevalence of depression, distributed by age and clinical characteristics and in-hospital outcomes among patients hospitalized with type 2 diabetes (T2DM) in Spain 2017–2023, according to sex; Table S3: In-hospital mortality among men and women hospitalized with type 2 diabetes, according to selected concomitant condition and to the presence of depression in Spain for the period 2017–2023; Figure S1: Joinpoint analysis of annual depression prevalence in women hospitalized with type 2 diabetes in Spain stratified by age groups (2017–2023); Figure S2: Joinpoint analysis of annual depression prevalence in men hospitalized with type 2 diabetes in Spain stratified by age groups (2017–2023).

## Abbreviations

T2DM	type 2 diabetes mellitus
RAE-CMBD	Registry of Specialized Care Activity–Minimum Basic Data Set
ICD-10	International Classification of Diseases, Tenth Revision
CCI	Charlson Comorbidity Index
LOHS	length of hospital stay
ICU	intensive care unit
IHM	in-hospital mortality
APC	annual percent change
NHANES	National Health and Nutrition Examination Survey
