# Supplementary material for: Trends in Depression Among Hospitalized Patients with Type 2 Diabetes in Spain (2017–2023): A Population-Based Analysis with a Focus on Sex Differences and In-Hospital Outcomes
Source: J Clin Med. 2025 Jun 1;14(11):3895. doi: 10.3390/jcm14113895 (PMC12156438; doi:10.3390/jcm14113895)
Supplement: Supplementary file 1 [file jcm-14-03895-s001.zip › English-Editing-Certificate-94617.pdf]

We certify that the following article

Trends in Depression Among Hospitalized Patients With Type 2 Diabetes in Spain (2017–2023): A Population-Based Analysis With a Focus on Sex Differences and In-Hospital Outcomes

Lucia Jiménez-Sierra, Natividad Cuadrado-Corrales, Valentín Hernández-Barrera, Rodrigo Jiménez-Garcia *, Ana López-de-Andres, Javier de Miguel-Diez, Andrés Bodas-Pinedo, José J Zamorano-León

has undergone English language editing by MDPI. The text has been checked for correct use of grammar and common technical terms, and edited to a level suitable for reporting research in a scholarly journal.

MDPI uses experienced, native English speaking editors. Full details of the editing service can be found at

► https://www.mdpi.com/authors/english.

MDPI

Basel, Switzerland

May 2025

AUTHORservices

english-94617
