# Supplementary material for: Trends in Depression Among Hospitalized Patients with Type 2 Diabetes in Spain (2017–2023): A Population-Based Analysis with a Focus on Sex Differences and In-Hospital Outcomes
Source: J Clin Med. 2025 Jun 1;14(11):3895. doi: 10.3390/jcm14113895 (PMC12156438; doi:10.3390/jcm14113895)

Figure S1.A. Joinpoint analysis of annual depression prevalence in women aged 40-64 years hospitalized with type 2 diabetes in Spain (2017-2023).

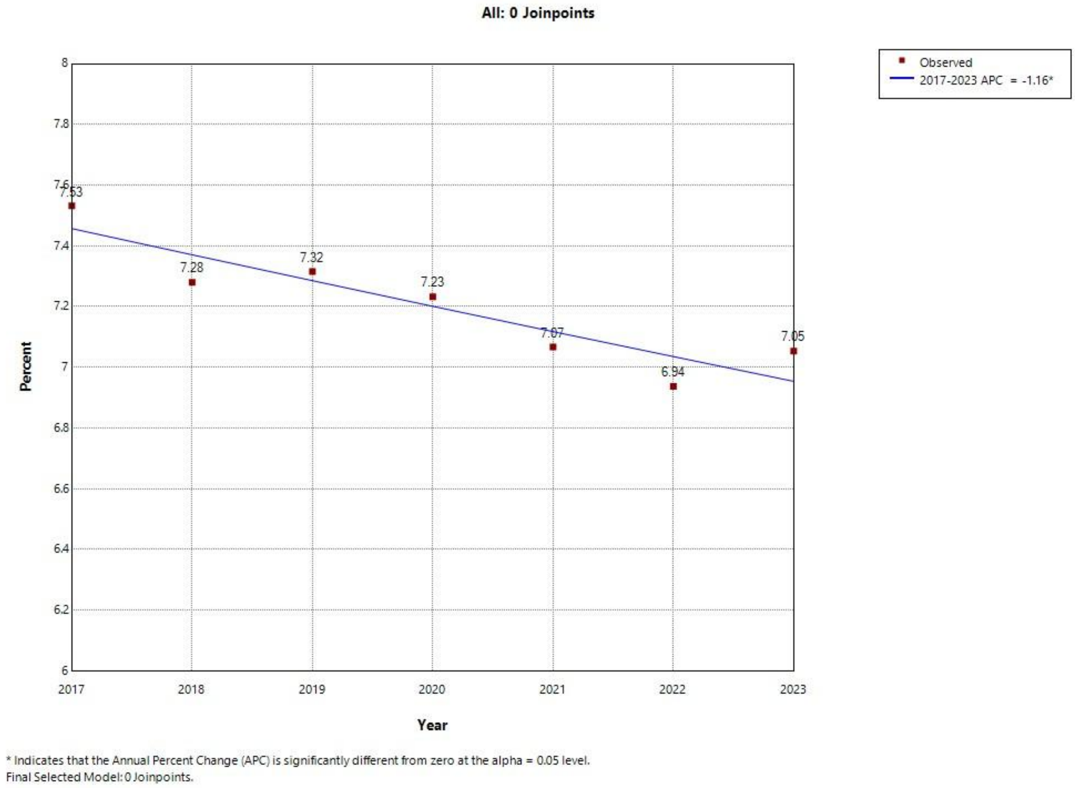

Figure S1.B. Joinpoint analysis of annual depression prevalence in women aged 65-74 years hospitalized with type 2 diabetes in Spain (2017-2023).

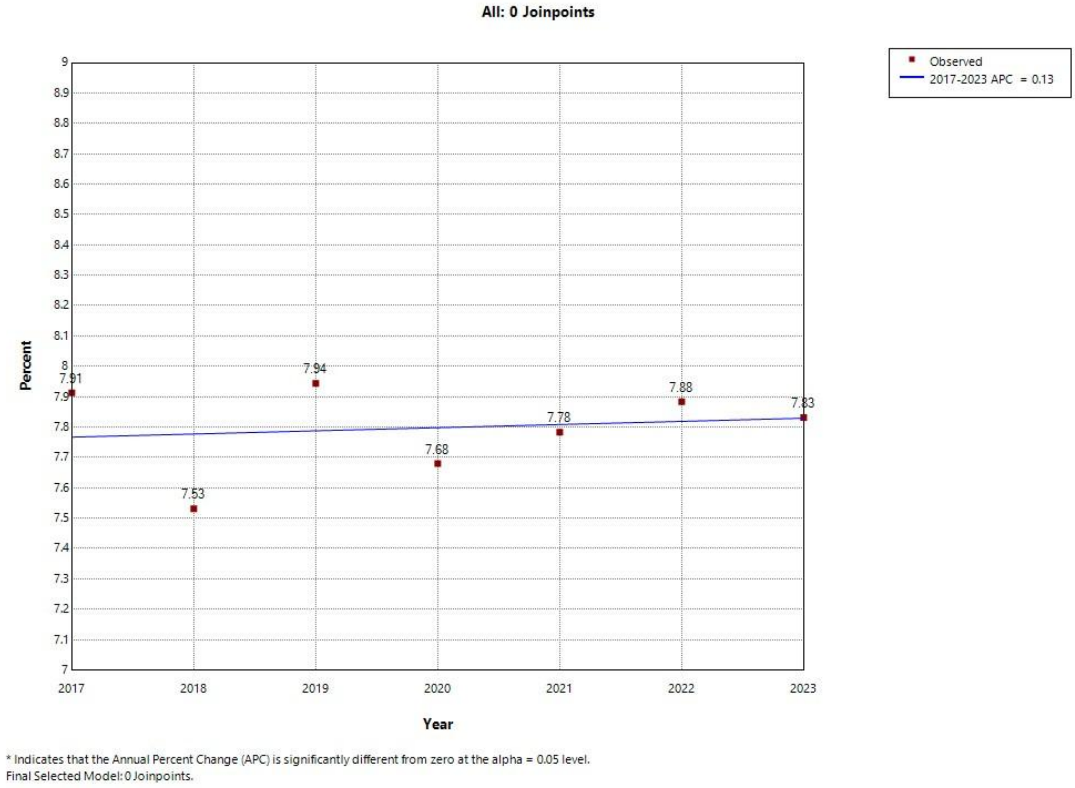

Figure S1.C. Joinpoint analysis of annual depression prevalence in women aged 75-84 years hospitalized with type 2 diabetes in Spain (2017-2023).

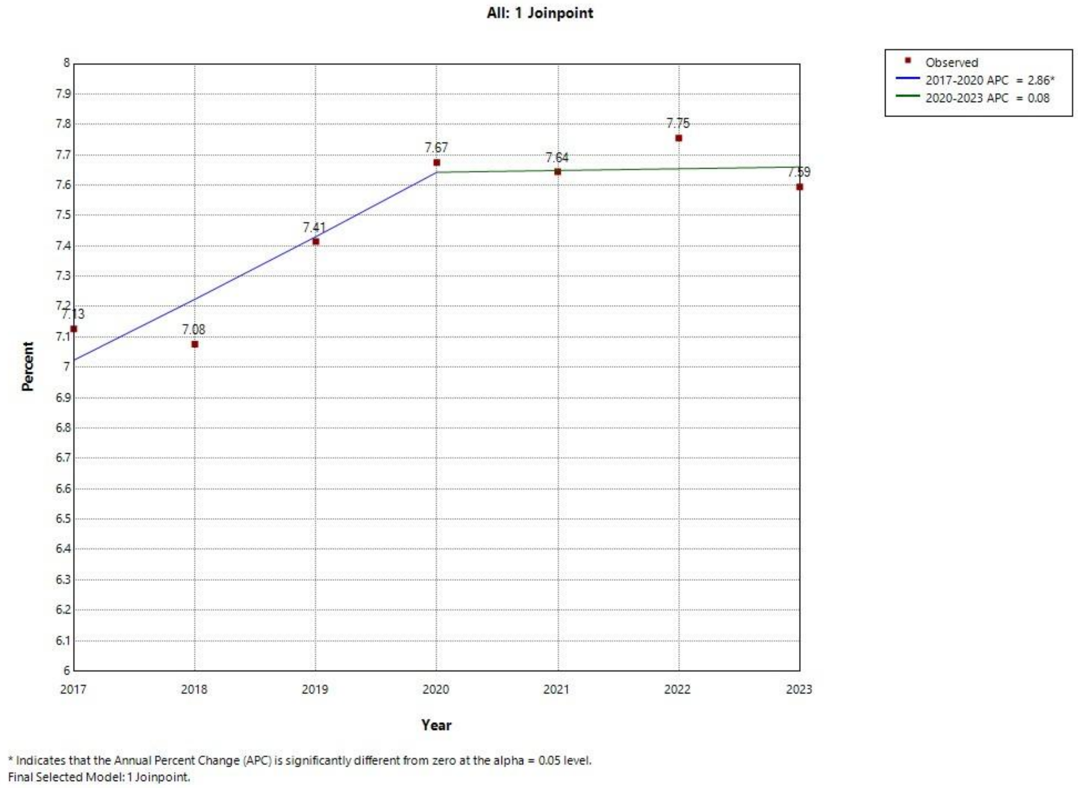

Figure S1.D. Joinpoint analysis of annual depression prevalence in women aged 85 years or more hospitalized with type 2 diabetes in Spain (2017-2023).

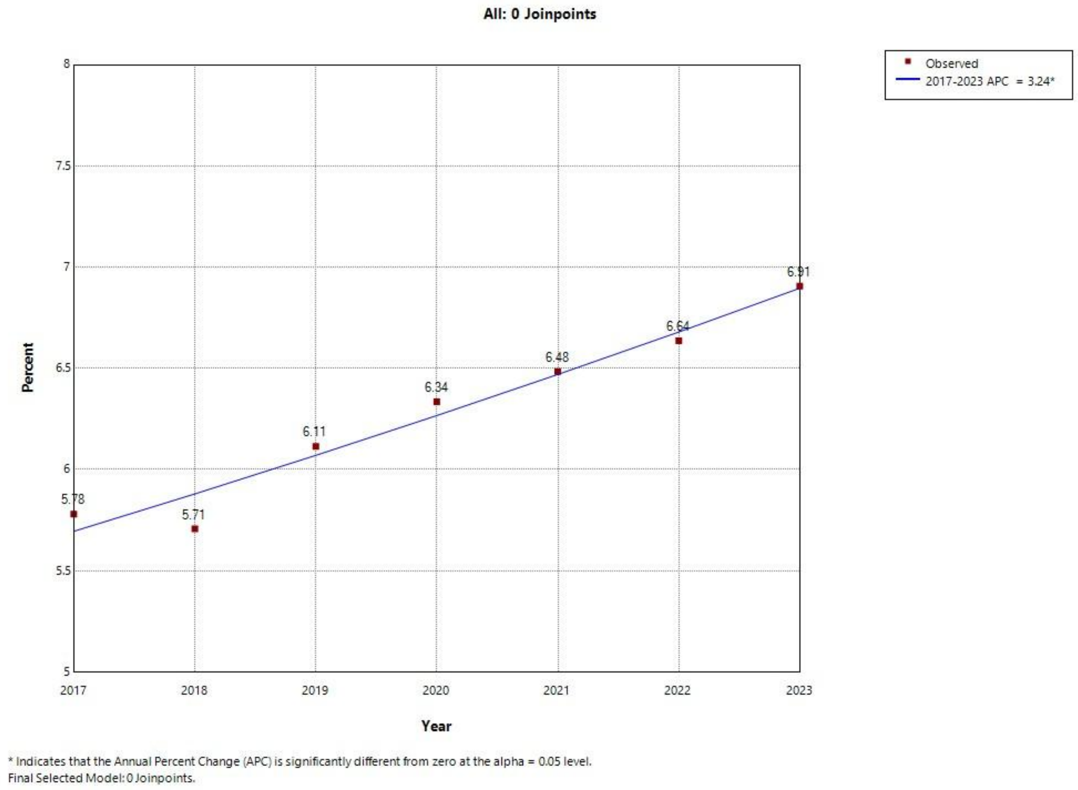

Supplement: Supplementary file 1 [file jcm-14-03895-s001.zip › Figure S1.pdf]
