# Supplementary material for: Trends in Depression Among Hospitalized Patients with Type 2 Diabetes in Spain (2017–2023): A Population-Based Analysis with a Focus on Sex Differences and In-Hospital Outcomes
Source: J Clin Med. 2025 Jun 1;14(11):3895. doi: 10.3390/jcm14113895 (PMC12156438; doi:10.3390/jcm14113895)

Figure S2.A. Joinpoint analysis of annual depression prevalence in men aged 40-64 years hospitalized with type 2 diabetes in Spain (2017-2023).

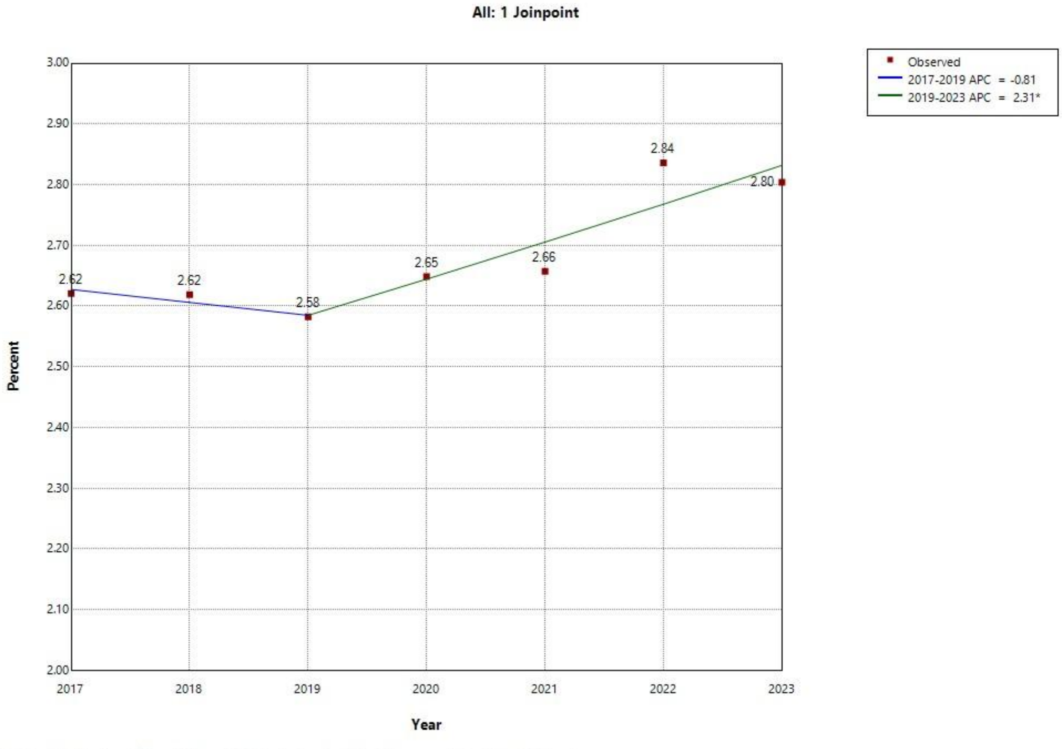

* Indicates that the Annual Percent Change (APC) is significantly different from zero at the alpha = 0.05 level.
Final Selected Model: 1 Joinpoint.

Figure S2.B. Joinpoint analysis of annual depression prevalence in men aged 65-74 years hospitalized with type 2 diabetes in Spain (2017-2023).

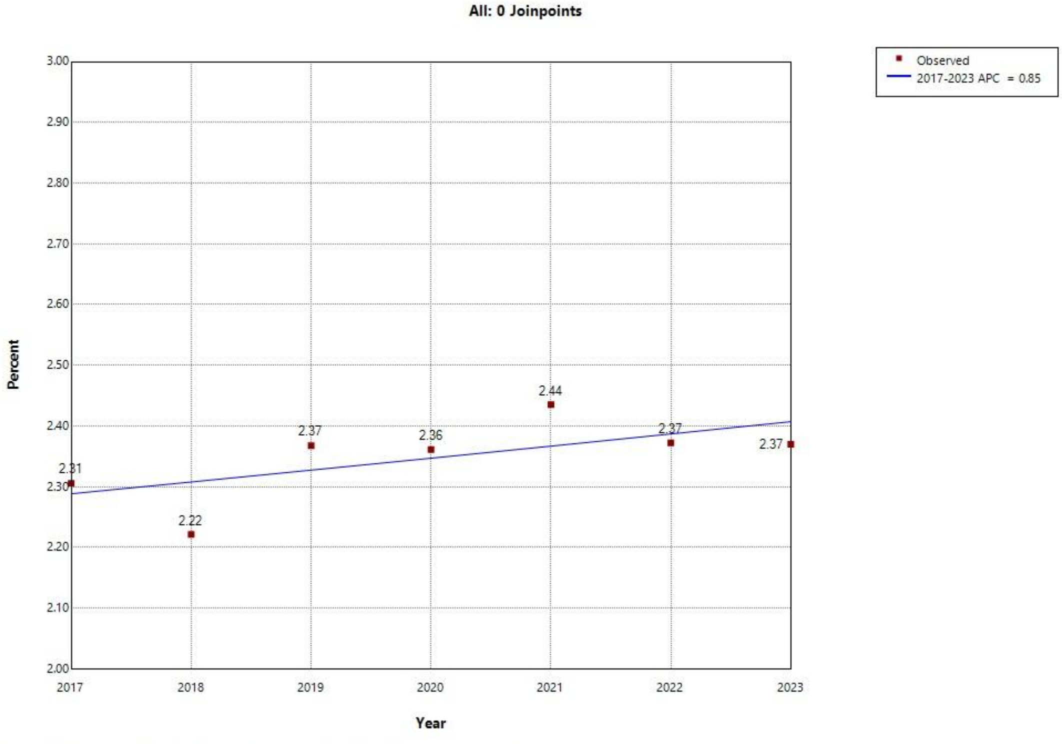

* Indicates that the Annual Percent Change (APC) is significantly different from zero at the alpha = 0.05 level.
Final Selected Model: 0 Joinpoints.

Figure S2.C. Joinpoint analysis of annual depression prevalence in men aged 75-84 years hospitalized with type 2 diabetes in Spain (2017-2023)

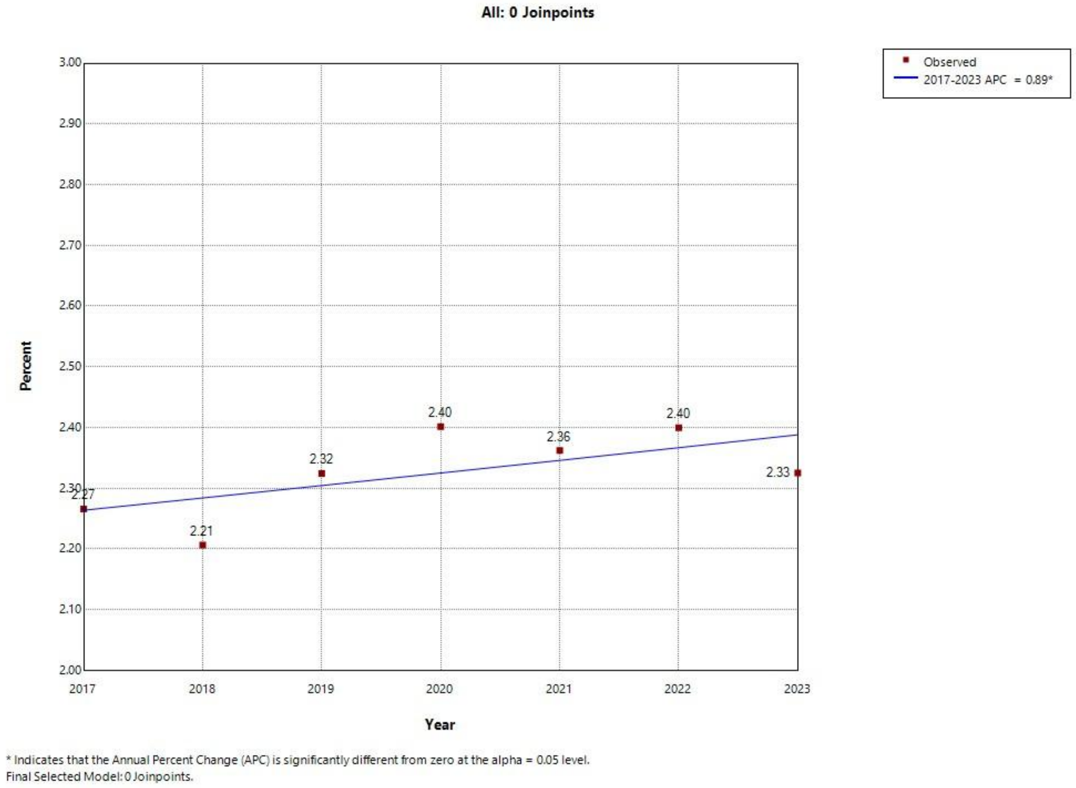

Figure S2.D. Joinpoint analysis of annual depression prevalence in men aged 85 years or more hospitalized with type 2 diabetes in Spain (2017-2023).

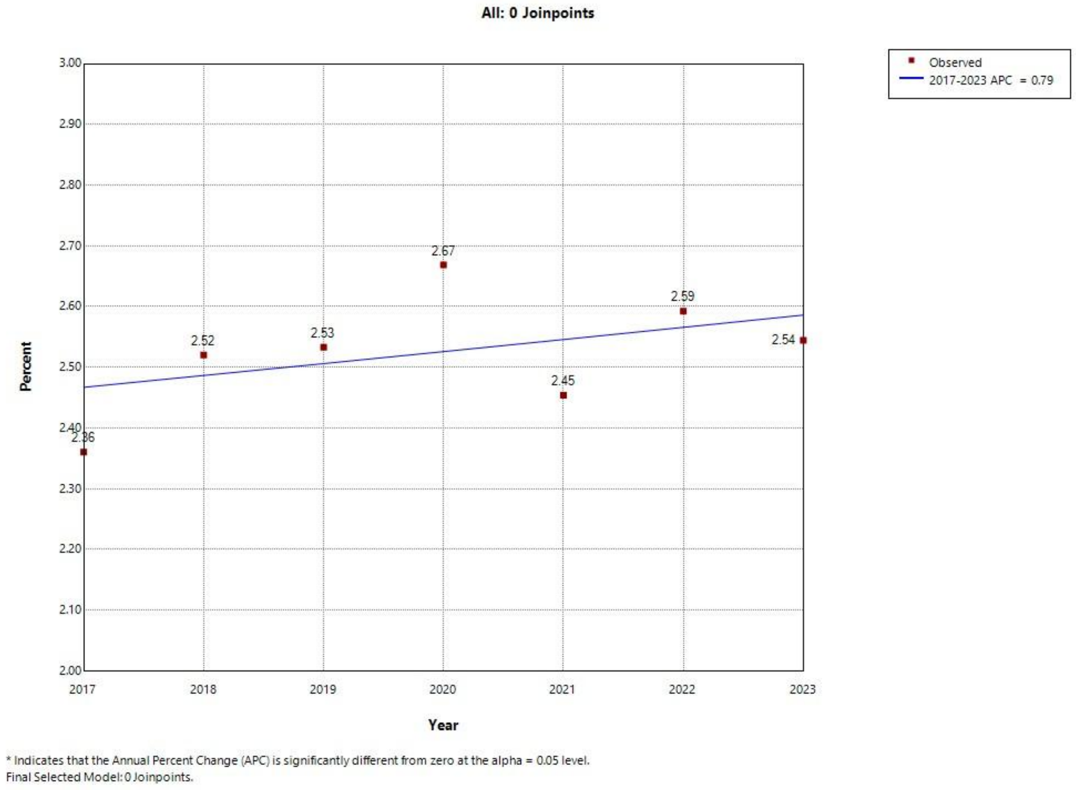

Supplement: Supplementary file 1 [file jcm-14-03895-s001.zip › Figure S2.pdf]
