# Supplementary material for: Trends in Depression Among Hospitalized Patients with Type 2 Diabetes in Spain (2017–2023): A Population-Based Analysis with a Focus on Sex Differences and In-Hospital Outcomes
Source: J Clin Med. 2025 Jun 1;14(11):3895. doi: 10.3390/jcm14113895 (PMC12156438; doi:10.3390/jcm14113895)
Supplement: Supplementary file 1 [file jcm-14-03895-s001.zip › Supplementary Tables.pdf]

Table S1. Diagnosis analyzed with their corresponding ICD10 codes.

**CONDITION**	**ICD 10 CODES**
Type 2 diabetes	E11
Depression	F32; F33
Hypoglycemia	E10.641; E10.649
Obesity	E66.09; E66.1 E66.3; E66.8 E66.9 E66.2; E66.01
Anxiety	F40; F41
Specific personality disorders	F60
Intentionally Self-Inflicted Injuries	X71 to X83
Suicide Attempt	T14.91
Alcohol-associated hospitalizations	E24.4; E51.2; F10; G31.2; G62.1; G72.1; I42.6; K29.20; K29.21 K70; K85.2; K86.0; T51.0; O35.4; R78.0; Y90; Z71.4
Tobacco use	F17; Z72.0; Z87.891; T65.2
All-cause dementia	G30; G31; F01
Alzheimer dementia	G30
Vascular dementia	F01
COVID 19	U071

Table S2. Prevalence of depression, distribution by age and clinical characteristics and in-hospital outcomes among patients hospitalized with type 2 diabetes (T2DM) in Spain 2017-2023, according to sex

		Women	Men	p
T2DM n		1909653	2688015	<0.001
2017 Depression, prevalence n (%)		18429(6.98)	8460(2.37)	<0.001
2018 Depression, prevalence n (%)		18558(6.8)	8888(2.35)	<0.001
2019 Depression, prevalence n (%)		19748(7.12)	9388(2.43)	<0.001
2020 Depression, prevalence n (%)		18128(7.2)	8963(2.49)	<0.001
2021 Depression, prevalence n (%)		19447(7.23)	9481(2.47)	<0.001
2022 Depression, prevalence n (%)		20757(7.29)	10175(2.52)	<0.001
2023 Depression, prevalence n (%)		21290(7.34)	10382(2.48)	<0.001
Age, mean (SD)		76.78(10.84)	72.69(11.28)	<0.001
Age groups	40-64 years, n (%)	19680(14.43)	16245(24.71)	<0.001
	65-74 years, n (%)	30977(22.72)	18850(28.67)	
	75-84 years, n (%)	48646(35.68)	19797(30.12)	
	≥85 years, n (%)	37054(27.17)	10845(16.5)	
CCI, mean (SD)		0.92(0.89)	1.09(0.97)	<0.001
Hypoglycemia, , n (%)		1765(1.29)	818(1.24)	0.348
Obesity, n (%)		31451(23.07)	9949(15.13)	<0.001
Anxiety, n (%)		2454(1.8)	979(1.49)	<0.001
Specific personality disorders, n (%)		1564(1.15)	1132(1.72)	<0.001
Intentionally Self-Inflicted Injuries, n (%)		993(0.73)	444(0.68)	0.186
Suicide Attempt, n (%)		82(0.06)	120(0.18)	0.026
Alcohol, n (%)		2490(1.83)	9424(14.34)	<0.001
Tobacco use, n (%)		15786(11.58)	26138(39.76)	<0.001
All-cause dementia, n (%)		16598(12.17)	6605(10.05)	<0.001
Alzheimer dementia n (%)		6289(4.61)	1769(2.69)	<0.001
Vascular dementia, n (%)		3051(2.24)	1508(2.29)	0.423
Covid 19, n (%)		5365(3.93)	2677(4.07)	0.138
Admission to ICU, n (%)		6124(4.49)	4262(6.48)	<0.001
LOHS, median (IQR)		6(7)	6(8)	0.077
IHM, n (%)		9835(7.21)	4732(7.2)	0.907

CCI: Charlson Comorbidity Index. ICU: intensive care unit. IHM: in-hospital mortality. LOHS: length of hospital stays. IQR Inter quartile range

Table S3. In-hospital mortality among men and women hospitalized with type 2 diabetes, according to selected concomitant condition and to the presence of depression in Spain for the period 2017-2023.

		IHM Women			IHM Men		
		**Without Depression**	**With Depression**	**p-value**	**Without Depression**	**With Depression**	**p-value**
Total, n (%)		153184 (8.63)	9835(7.21)	<0.001	197038 (7.51)	4732(7.2)	0.002
Age, mean (SD)		82.92(9.37)	82.19(9.05)	<0.001	78.13(10.16)	78.00(10.17)	0.378
Age groups	40-64 years, n (%)	7786(3.07)	488(2.48)	<0.001	21370(3.62)	541(3.33)	0.047
	65-74 years, n (%)	17962(4.9)	1281(4.14)	<0.001	44562(5.68)	1043(5.53)	0.375
	75-84 years, n (%)	48010(7.96)	3435(7.06)	<0.001	70414(8.47)	1683(8.5)	0.895
	≥85 years, n (%)	79426(14.44)	4631(12.5)	<0.001	60692(14.52)	1465(13.51)	0.003
CCI, mean (SD)		1.35(0.95)	1.27(0.94)	<0.001	1.55(1.02)	1.46(1.03)	<0.001
Hypoglycemia, , n (%)		3228(13.76)	167(9.46)	<0.001	3704(14.37)	94(11.49)	0.021
Obesity, n (%)		22987(6.7)	1781(5.66)	<0.001	18117(5.05)	444(4.46)	0.008
Anxiety, n (%)		6150(6.23)	125(5.09)	0.021	2672(5.9)	34(3.47)	0.002
Specific personality disorders, n (%)		331(4.72)	47(3.01)	0.003	357(4.97)	44(3.89)	0.115
Intentionally Self-Inflicted Injuries, n (%)		835(6.85)	41(4.13)	0.001	1023(8.87)	43(9.68)	0.557
Suicide Attempt, n (%)		1(0.48)	3(3.66)	0.077	15(4.17)	4(3.33)	0.686
Alcohol, n (%)		1746(7.35)	112(4.5)	<0.001	22136(7.42)	553(5.87)	<0.001
Tobacco use, n (%)		8314(4.83)	654(4.14)	<0.001	67696(6.38)	1568(6)	0.013
All-cause dementia, n (%)		23553(14.36)	1813(10.92)	<0.001	19783(14.44)	694(10.51)	<0.001
Alzheimer dementia n (%)		12321(16)	840(13.36)	<0.001	8062(17.31)	226(12.78)	<0.001
Vascular dementia, n (%)		4647(15.46)	340(11.14)	<0.001	4709(15.15)	186(12.33)	0.003
Covid 19, n (%)		10423(14.88)	707(13.18)	0.001	16015(15.42)	362(13.52)	0.007

CCI: Charlson Comorbidity Index.
